# P16 as a marker of carcinoma in effusions and peritoneal washing

**DOI:** 10.1186/s12885-020-6670-5

**Published:** 2020-03-17

**Authors:** Fabiana Pirani Carneiro, Rivadávio Fernandes Amorim, Marcos de Vasconcelos Carneiro, Tercia Maria Mendes Lousa de Castro, Leonora Maciel de Souza Vianna, Gustavo Henrique Soares Takano, Andersen Charles Daros, Isabela Peres, Selma Aparecida Souza Kuckelhaus, Andrea Barretto Motoyama

**Affiliations:** 1 Pathological Anatomy Center of University Hospital of Brasilia, Via L2 Norte, SGAN 604/605, Brasília DF, 70840-050 Brazil; 2 Pathology Department of Brasília University, Brasília, Brazil; 3 Catholic University of Brasilia, Brasília, Brazil; 4 Morphology Department of Brasília University, Brasília, Brazil

**Keywords:** Cytology, Immunocytochemistry, p16, effusion, Carcinoma

## Abstract

**Background:**

Considering the potential of p16 as a marker for diagnosis, prognosis and therapeutic response, the aim of this study was to assess its presence, via immunocytochemistry, in metastatic carcinoma of different primary sites and histological types obtained from effusions and peritoneal washings. A total of 118 samples including 85 of metastatic carcinoma and 33 samples of benign effusion/peritoneal washing were prepared by the plasma/thromboplastin method. Immunocytochemistry reactions were performed on cell block sections using antibodies against p16, claudin-4, MOC-31, calretinin, HBME and CD68.

**Results:**

P16 overexpression was observed in 88.23% of all carcinoma samples. All cervix adenocarcinoma samples showed p16 overexpression. Overexpression in adenocarcinomas of ovary, lung and breast was observed in 93.75, 93.10 and 75% of the samples, respectively. Overexpression was observed in all different histological types analyzed: small cell carcinoma (lung), squamous cell carcinoma (cervical) and urothelial carcinoma (bladder). The specificity of p16 for carcinoma detection was of 96.96%.

**Conclusion:**

Overexpression of p16 was observed in most metastatic carcinoma, from different primary sites and histological types, obtained from effusions and peritoneal washings. Due to its high frequency of overexpression in metastatic carcinoma, p16 may play a possible role in tumor progression and it may be considered as a complementary diagnostic marker depending on histological type and primary site of carcinoma.

## Background

The use of markers for diagnosis, prognosis and therapeutic response may be necessary in follow up of patients with metastatic carcinoma obtained from effusion/peritoneal washing. The p16 tumor suppressor gene is a member of the INK4 (Cyclin-dependent Kinase 4 Inhibitor) class of cell cycle inhibitors [1]. The p16 protein binds to cyclin-dependent kinases 4 and 6 and maintains the retinoblastoma (RB) gene product in its hypophosphorylated state, which in turn binds to E2F transcription factor and prevents cell cycle progression [1]. In human papillomavirus (HPV) related tumors, integration of the virus into the host cell genome leads to production of the E7 viral oncoprotein that functionally inactivates pRb, preventing it from binding to the E2F transcription factor [2]. As a consequence, an increased expression of p16 occurs in both the nucleus and cytoplasm, which can be detected by immunohistochemistry [3]. In non-HPV related tumors, p16 expression may be either reduced due to p16 gene deletions, mutations or epigenetic silencing or increased due to RB loss of function (gene deletions, point mutations, functional mutations or other mechanisms of Rb pathway deregulation) [2].

The expression of p16 is variable in carcinomas from different sites of origin and between histological types of the same site. Small cell lung carcinoma is characterized by high levels of p16 while lung adenocarcinoma shows low levels [4, 5]. In breast, p16 is overexpressed in basal-like carcinomas, and can be used as a marker to distinguish this from the other subtypes of breast carcinomas [6]. Most ovarian/tubal high-grade serous carcinomas are diffusely positive for p16, while low-grade serous, endometrioid, clear cell and mucinous carcinomas are usually negative or focally positive [7, 8].

The prognostic value of p16 protein overexpression is also variable between carcinomas from different sites of origin and between histological types of the same site. High p16 expression in breast carcinoma correlates with unfavorable prognostic factors such as poor overall survival, poor disease-free survival, ER (estrogen receptor) and PR (progesterone receptor) negativity and an increased risk of relapse cancer [9]. The role of p16INK4a in the tumorigenesis of lung cancer and its biological contributions to a poorer prognosis remain unclear [10]. In ovarian cancer, association of p16 expression with prognosis is different across ovarian carcinoma histological types; absence of p16 expression predicts shorter survival for low-grade serous carcinoma while no survival associations are observed for mucinous carcinomas or high-grade serous carcinomas [11].

P16 is also a potential biomarker for predicting the response with chemo (radio) therapy for cancer from different primary sites such as breast and esophageal cancer [12, 13].

Considering its potential as a marker for diagnosis, prognosis and therapeutic response and since it has not been yet reported in the literature, the aim of this study was to assess immunoreactivity for p16 in metastatic carcinoma from different primary sites and histological types obtained from effusions and peritoneal washings.

## Methods

A total of 118 samples (pleural effusion *n* = 59, peritoneal effusion *n* = 32, pericardial effusion *n* = 9 and peritoneal washing *n* = 18), including 85 of metastatic carcinoma and 33 of benign effusion/peritoneal washing, were analyzed at the Pathology Department of the Brasilia University Hospital, Brazil. This study was approved by the Human Ethics Review Committee of Brasilia University. All subjects provided written consent to participate in the study. The diagnoses of benign effusions/peritoneal washings were as follows: cystadenoma (*n* = 7), pleuritis (*n* = 3), teratoma (*n* = 2), pericarditis (*n* = 2), leiomyoma (*n* = 2), endometriosis (*n* = 2), pneumonia (*n* = 2), cholecystitis (*n* = 2), cardiac insufficiency (*n* = 1), pancreatic cyst (*n* = 1), benign tumor of Brenner (*n* = 1), fibroma (*n* = 1), nephrotic syndrome (*n* = 1), Crohn’s disease (*n* = 1), rheumatoid arthritis (*n* = 1), colon polyposis (*n* = 1), abscess (*n* = 1), peritonitis (*n* = 1) and eosinophilic ascites (*n* = 1).

This was an observational and cross-sectional study. During the period of 2015–2019, samples were selected from 539 fluid samples and 256 peritoneal washings. Diagnosis was performed on cytological features, clinical history, imaging studies, corresponding histological results, and immunocytochemical findings. Only samples with at least 10 carcinoma cells positive for claudin-4 or MOC-31 in cell block sections were included.

The expression of p16 in histological samples (*n* = 15) of primary sites was also analyzed for comparison with the expression of p16 in the respective metastasis samples (cell block): lung, *n* = 7; ovary, *n* = 5 and breast, *n* = 3.

All samples were freshly prepared and no fixatives or preservative solutions were used. Cell block preparation and immunocytochemistry was performed as previously described [14], using the plasma-thromboplastin method. In brief, samples were centrifuged and 100 μl of plasma and 100 μl of thromboplastin (Stago®, Asnières sur Seine, France) were added onto the cell pellet. The clots were formalin-fixed, submitted to usual histological processing, and sections mounted on previously silanized slides. These were stained with hematoxylin-eosin and used for immunocytochemistry.

Prior to exposure to the primary antibodies, samples were submitted to antigen retrieval in citrate buffer pH 6.0 in a waterbath at 95–99 °C for 45 min. For blockade of endogenous tissue peroxide, the slides were immersed in 3% H_2_O_2_ solution at room temperature for 30 min, and thoroughly washed with phosphate buffered saline (PBS). Incubation with primary antibodies (shown in Table 1) was performed overnight at 4 °C. After a 30-min-incubation with secondary antibody at room temperature, positive cells were marked with the streptavidin-peroxidase complex (Kit REVEAL - Biotin-Free Polyvalent DAB - Spring Bioscience®, CA, USA) and the reactions were developed using a diaminobenzidine chromogen solution (kit REVEAL - Biotin-Free Polyvalent DAB - Spring Bioscience®, California, USA). Harris hematoxylin was used for counterstaining. Positive and negative controls were used for each primary antibody, according to the manufacturer recommendation.

**Table 1:** Antibodies used for immunocytochemistry

Antibody	Source	Clone	Dilution
P16	ZETA	G175–405	1:50
Epithelial related antigen	DAKO	MOC-31	1:200
Claudin-4	NOVEX	3E2C1	1:200
Calretinin	DAKO	DAK-calret1	1:50
Mesothelial cell	CELL MARQUE	HBME1	1:50
CD68	BIOCARE	KP1	1:100

Positive reaction was defined as p16 expressed in the nucleus and/or cytoplasm of tumor cells. For claudin-4 and MOC-31, positive staining was defined as a brown stain in cell membrane. Expression of claudin-4, MOC-31 and p16 was evaluated by calculating a total immunostaining score (TIS) as the product of a proportion score (PS) and an intensity score (IS). The PS describes the estimated fraction of positively stained tumor cells (0, none; 1, < 10% of cells; 2, 10–50% of cells; 3, 51–80% of cells; 4, > 80% of cells). The IS refers to the estimated staining intensity as compared to control, varying from 0, 1, 2, and 3 to no staining’, ‘weak’, ‘moderate’ and ‘strong’, respectively. The TIS then ranges from 0 to 12 with only nine possible values (that is, 0, 1, 2, 3, 4, 6, 8, 9 and 12), as it is derived from TIS=PS x IS. By this score system, four subgroups were defined: no expression, TIS 0; weak expression, TIS 1–4; moderate expression, TIS 6, 8; intense expression, TIS 9, 12. ‘Overexpression’ has been previously defined as a TIS > 4 (moderate and intense expression) [15]. For calretinin, mesothelial cell and CD68 positive staining was defined as a strong brown stain in more than 1% of cells in the cytoplasm and nucleus (calretinin), membrane (HBME) and cytoplasm (CD68).

Statistical analysis was performed with Graphpad Prism 4 (GraphPad Software, San Diego, CA). Kruskal-Wallis test was used to compare medians of TIS values of the markers (for claudin-4, MOC-31 and p16). Spearman’s correlation test was used to assess the correlation between TIS values of claudin-4, MOC-31 and p16.

## Results

### Claudin-4

Overexpression was observed in all carcinoma samples (*n* = 85), including adenocarcinomas from different primary sites and carcinomas of different histological types such as squamous cell carcinoma (cervical), small cell carcinoma (lung), and urothelial carcinoma (bladder). The TIS values in samples with overexpression ranged from 6 to 12, corresponding to moderate and intense expression (Table 2). Overexpression was detected in mesothelial cells from 6.06% (2/33) of benign effusion/peritoneal washing samples. The TIS values in these benign samples with overexpression was 6, corresponding to moderate expression. Weak cytoplasmic expression was observed in macrophages.

**Table 2:** Overexpression of claudin -4, MOC-31 and p16 according to primary sites of carcinoma and histological type

Carcinoma	Claudin-4 Overexpression n (TIS)		MOC-31 Overexpression n (TIS)		P16 Overexpression n (TIS)	
Adenocarcinomas	absence	presence	absence	presence	absence	presence
Lung (*n* = 29)	0	29 (6–12)	0	29 (6–12)	2 (1,4)	27 (6–12)
Ovary (*n* = 16)	0	16 (8,12)	0	16 (8,12)	1 (1)	15 (6–12)
Breast (*n* = 16)	0	16 (6–12)	0	16 (6–12)	4 (2,4)	12 (8–12)
Stomach (*n* = 6)	0	6 (6–12)	0	6 (6–12)	2 (2,4)	4 (8,9)
Colon (*n* = 5)	0	5 (6–12)	0	5 (6–12)	0	5 (6–12)
Biliary tract (*n* = 3)	0	3 (8,12)	0	3 (8,12)	0	3 (6)
Cervix (*n* = 2)	0	2 (8,12)	0	2 (8,12)	0	2 (12)
Pancreas (*n* = 1)	0	1 (12)	0	1 (12)	1 (2)	0
Endometrium (*n* = 1)	0	1 (12)	0	1 (12)	0	1 (12)
Kidney (*n* = 1)	0	1 (12)	0	1 (12)	0	1 (12)
Unkonwn (*n* = 2)	0	2 (8,12)	0	2 (8,12)	0	2 (12)
Subtotal Adenocarcinomas	0	82 (6–12)	0	82 (6–12)	10 (1–4)	72 (6–12)
Other histological types						
Squamous carcinoma (cervix) (*n* = 1)	0	1 (9)	1 (1)	0	0	1 (12)
Urothelial carcinoma (bladder) (*n* = 1)	0	1 (12)	1 (4)	0	0	1 (8)
Small cell carcinoma (lung) (*n* = 1)	0	1 (6)	0	1 (12)	0	1 (8)
Total (*n* = 85)	0	85	2	83	10	75

### MOC-31

Overexpression was observed in 97.64% (83/85) of all carcinoma samples. All adenocarcinoma samples from different primary sites showed overexpression. In carcinomas of different histological types, overexpression was observed only in small cell carcinoma (lung) (Table 2). The TIS values in carcinoma samples with overexpression ranged from 6 to 12, corresponding to moderate and intense expression (Table 2). Overexpression was detected in mesothelial cells from 18.18% (6/33) of benign effusion/peritoneal washing samples. The TIS values in these benign samples with overexpression was 6, corresponding to moderate expression (Fig. 2). No expression was observed in macrophages.

### P16

**Fig. 1:**
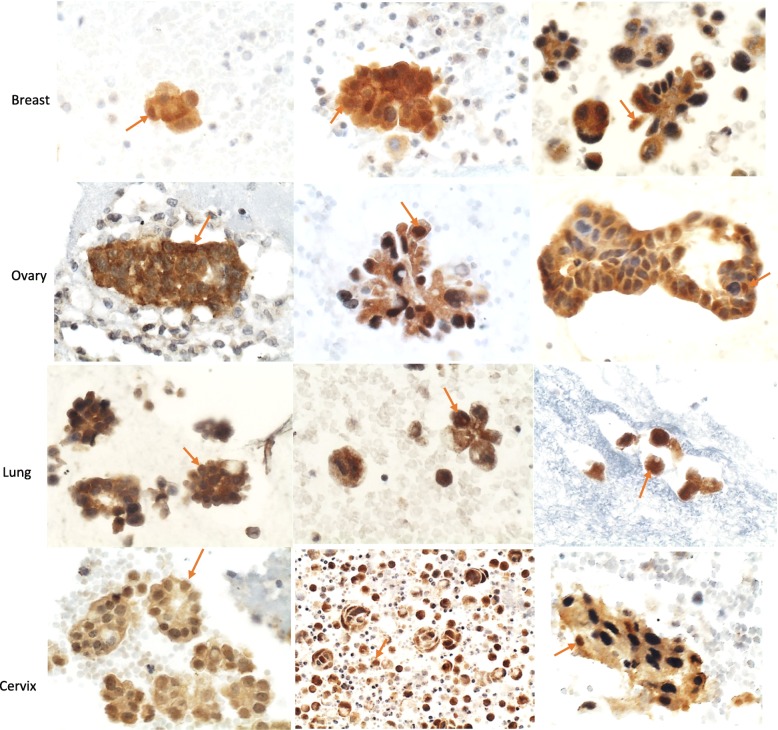
P16 overexpression in metastatic carcinomas of breast, ovary lung and of cervix (cell block of effusion/peritoneal washing, immunocytochemistry, 400x). Nuclear expression (orange arrow)

Overexpression was observed in 88.23% (75/85) of all carcinoma samples. All cervix adenocarcinoma samples showed p16 overexpression. (Table 2, Fig. 1). Overexpression in adenocarcinomas of ovary, lung and breast was observed in 93.75, 93.10 and 75% of the samples, respectively (Table 2, Fig. 1). Overexpression was observed in all different histological types analyzed: small cell carcinoma (lung), squamous cell carcinoma (cervical) and urothelial carcinoma (bladder) (Table 2). The TIS values in carcinoma samples with overexpression ranged from 6 to 12, corresponding to moderate and intense expression (Table 2). The intensity of nuclear expression was proportional to cytoplasmic expression (Fig. 1). Overexpression was detected in mesothelial cells from 3.03% (1/33) of benign effusion/peritoneal washing samples. The TIS value in this benign sample with overexpression was 12, corresponding to intense expression. Weak cytoplasmic expression was occasionally observed in macrophages.

**Fig. 2:**
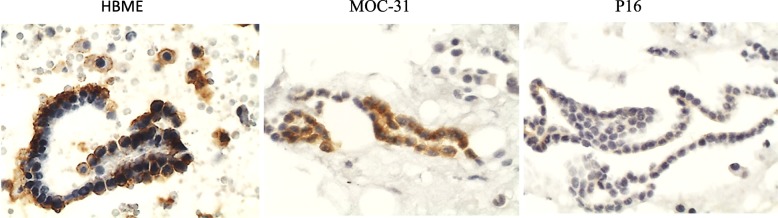
P16 overexpression in metastatic (cell block) and in respective primary carcinoma (histology) of ovary and lung. Nuclear expression (orange arrow)

There was p16 overexpression in the primary site (histology) and in respective metastasis (cell block) in 93.3% (14/15) of the patients (Fig. 2). In one case of breast carcinoma, p16 overexpression was observed only in the metastasis (cell block) sample.

### Comparison and correlation between TIS values of markers

The mean (SD) of TIS values was, respectively, 10.90 (2.08), 10.87 (2.37) and 9.92 (3.30) for claudin-4, MOC-31 and p16. The median of TIS values was 12 for all these markers; and no significant difference was observed between TIS values medians (Kruskall-Wallis, *p* > 0.05) for the markers.

There was a positive correlation between TIS values for claudin-4 and MOC-31 (Spearman, *r* = 0.84). There was no correlation between TIS values for p16 and those for claudin-4 and MOC-31 (Spearman, *r* = 0.23 and *r* = 0.17, respectively).

### HBME, calretinin and CD68

**Fig. 3:**
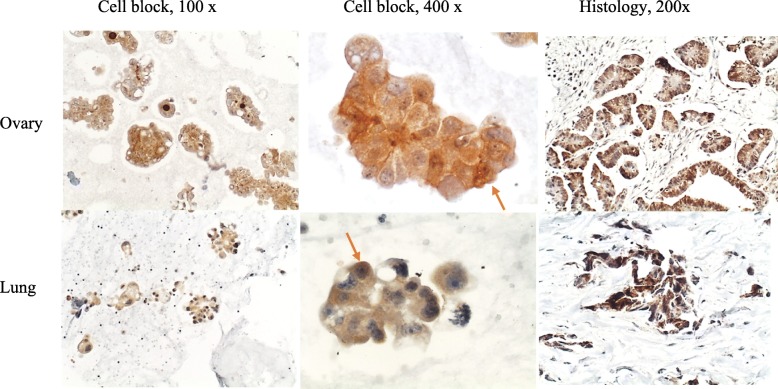
Sheets of normal mesothelial cell in peritoneal washing of a patient with endometriosis. Normal expression of HBME, overexpression (moderate expression, TIS = 6) of MOC-31 and no overexpression (weak expression) of p16. (Cell block, immunocytochemistry, 400x)

HBME was positive in mesothelial cells arranged in sheets or strips, in cells of ovary carcinoma and in cells of lung carcinoma of some samples (Fig. 3). Calretinin was positive in isolated reactive mesothelial cells and CD68 in macrophages.

## Discussion

Immunostaining for p16 has been applied in both cytology and histology specimens. Noteworthy, there is no general consensus for establishing criteria of p16 positivity. Consequently, lack of standardization makes it difficult to analyze the clinical implications of p16 immunostaining. This is especially a problem in cytology, since the histological criteria of p16 positivity (negative, focal, diffuse) are now widely accepted [16, 17]. Other features of p16 staining have not been analyzed systematically, e.g. nuclear versus cytoplasmic staining or the intensity of staining [16].

The immunoreactivity of anti-p16 antibody was evaluated and compared with the expression of claudin-4 and MOC-31 by using total immunostaining score (TIS), which is the product of the proportion score and the intensity score. This score has been previously used to evaluate the expression of p16 and EpCAM in histological specimens [9, 14, 15]. Here, this score was applied to enable comparison between p16 expression with those of MOC-31 (anti-EpCAM clone) and claudin-4. Whereas weak expression of these markers can be observed in normal cells, only ‘overexpression’ (moderate and intense expression, TIS > 4) was considered for comparison analysis.

The sensitivity for metastatic carcinoma detection from effusion/peritoneal washing was 100, 97.64 and 88.23% for claudin-4, MOC-31 and p16, respectively. Claudin-4 was used as a reference for comparison with the p16 results since it is considered as the most sensitive marker to distinguish adenocarcinomas from reactive and malignant mesothelial cells in cytology of effusions [18, 19]. MOC-31 is also a useful diagnostic marker with high sensitivity and specificity for differentiating malignant from benign effusions. However, this marker may not be expressed in some carcinomas of histological types other than adenocarcinomas, which may result in false negative diagnoses [18].

Cell block was used as method for cytological preparation since, comparing to Cytospin and ThinPrep samples, demonstrates more similarity in morphology and immunohistotochemistry findings to surgical pathology specimens [20]. Here, the plasma/tromboplastin method was chosen to prepare cell blocks because in comparison with other methods, it is easy to perform, of low cost and results in high cellularity section, homogeneous cell distribution and clear marker expression [21].

To the best of our knowledge, this is the first study that evaluated p16 expression in metastatic carcinoma obtained from effusions. Most of the samples of adenocarcinomas showed p16 overexpression. The other types of carcinomas, such as small cell carcinoma (lung), squamous cell carcinoma (cervical) and urothelial carcinoma (bladder) also showed p16 overexpression. These high levels of p16 in metastatic carcinomas from effusion/peritoneal washing suggest a possible inactivation / dysregulation of the Rb tumor suppressor gene and consequent overexpression of p16, which arises during tumor progression.

The most frequent primary sites of metastatic adenocarcinomas observed in this study were ovary, lung and breast. Most samples of carcinoma with primary site in ovary were high-grade serous carcinomas and positive for p16. This finding is in accordance with previous studies in which most ovarian high-grade serous carcinomas were diffusely positive (homogenous staining) for p16 in histopathological samples [7, 8]. The carcinoma sample with primary site in ovary that was negative for p16 was the clear cell carcinoma histological type.

Overexpression of p16 was observed in most metastatic lung adenocarcinomas samples of the present study. Previously, in primary non-small cell lung carcinomas, its expression was observed in 40.8% of the samples and was more frequent in squamous cell carcinoma than in adenocarcinomas [22]. In the same study, HPV DNA was detected in 1.5% of the samples, lack of Rb staining was observed 27.4% of the samples and the authors showed an inverse correlation between p16 and Rb protein.

About 39% of the invasive breast carcinoma of ductal type showed p16 overexpression (moderate and intense expression) in a previous study in which 1042 histological samples obtained from primary site were analyzed with a similar score used in present study [9]. In this previous study, the authors also concluded that patients with strongly p16-positive cancers had a 2.5-fold increased risk of death and more than three-fold increased risk of disease recurrence as compared with those with p16-negative cancers. In comparison with this previous study, the metastatic breast carcinoma samples of the current study showed a higher percentage (75%) of p16 overexpression.

With respect to the carcinomas of other histological types, p16 expression is very frequent in small cell lung carcinoma in biopsies [5]. As expected, the small cell lung carcinoma sample of the present study was also positive for p16. P16 overexpression in squamous carcinoma of cervical origin was also expected because of its association with HPV infection. Overexpression of p16 is also frequent in urothelial carcinoma and may occur in the absence of demonstrable HPV DNA [23].

The specificity for carcinoma detection was 93.93, 81.81 and 96.96% for claudin-4, MOC-31 and p16, respectively. Only overexpression (moderate and intense expression, or TIS > 4) was considered for comparison analysis. In fact, due to the difficulty in differentiating reactive mesothelial cells from malignant epithelial cells, at least two mesothelial cells markers, such as anti-calretinin and anti-mesothelial cell, are required for carcinoma screening in effusions. In the present study, these markers were used to assess the specificity of p16 for detection of carcinoma in effusions/peritoneal washings. Like MOC31 and claudin-4, p16 is not specific for malignant epithelial cells. However, p16 showed a higher specificity than these markers for detection of carcinoma.

When the hypothesis is mesothelioma, fluorescent in situ hybridization (FISH) is a more reliable method of assessing p16 status in mesothelial cells [24, 25]. Combined BRCA1-associated protein 1 immunohistochemistry/p16 FISH testing (detection of p16 homozygous deletion) is a highly specific method of diagnosing malignant mesotheliomas [24, 25].

Similarly to claudin-4, a weak cytoplasmic expression in macrophages was occasionally observed by using p16 in the present study. In this way, as some malignant epithelial cells are arranged in isolation mimicking macrophages, use of CD68 and adequate analysis of the morphological aspect and of the intensity and staining pattern (nucleus and/or cytoplasm) should be performed to avoid false positive results.

## Conclusion

Overexpression of p16 was observed in most metastatic carcinoma from different primary sites and histological types obtained from effusions and peritoneal washings. Due to its high frequency of overexpression in metastatic carcinoma, p16 may play a possible role in tumor progression, and it may be considered as a complementary diagnostic marker depending on histological type and primary site of carcinoma.

## Data Availability

All data generated or analyzed during this study are included in this published article.

## Abbreviations

ER: Estrogen receptor
HPV: Human papillomavirus
INK4: Cyclin-dependent Kinase 4 Inhibitor
IS: Intensity score
PBS: Phosphate buffered saline
PR: Progesterone receptor
pRB: Retinoblastoma protein
PS: Proportion score
TIS: Total immunostaining score
